# Validation of the patient health questionnaire-9 and the generalized anxiety disorder-7 in Lithuanian student sample

**DOI:** 10.1371/journal.pone.0263027

**Published:** 2022-01-27

**Authors:** Aiste Pranckeviciene, Ausra Saudargiene, Julija Gecaite-Stonciene, Vilma Liaugaudaite, Inga Griskova-Bulanova, Dovile Simkute, Rima Naginiene, Laurynas Linas Dainauskas, Gintare Ceidaite, Julius Burkauskas

**Affiliations:** 1 Laboratory of Behavioral Medicine, Neuroscience Institute, Lithuanian University of Health Sciences, Palanga - Kaunas, Lithuania; 2 Laboratory of Biophysics and Bioinformatics, Neuroscience Institute, Lithuanian University of Health Sciences, Kaunas, Lithuania; 3 Department of Informatics, Vytautas Magnus University, Kaunas, Lithuania; 4 Department of Neurobiology and Biophysics, Institute of Biosciences, Vilnius University, Vilnius, Lithuania; 5 Laboratory of Toxicology, Neuroscience Institute, Lithuanian University of Health Sciences, Kaunas, Lithuania; University of São Paulo, BRAZIL

## Abstract

**Background:**

The Patient Health Questionnaire—9 (PHQ-9) and the Generalized Anxiety Disorder Questionnaire– 7 (GAD-7) are short screening instruments used for detection of depression and anxiety symptoms in various settings, including general and mental health care as well as the general population. The aim of this study is to evaluate psychometric properties and factorial structure of the PHQ-9 and the GAD-7 in a sample of Lithuanian university students.

**Methods:**

1368 students (mean age 22.5±4.8) completed the PHQ-9 and the GAD-7 questionnaires online; after the completion of the survey, students were asked to provide phone contact for an additional interview. Eligible students were approached later by trained interviewers and completed The Clinical Interview Schedule-Revised for assessment of depressive and anxiety disorders.

**Results:**

Results showed that the PHQ-9 and the GAD-7 are reliable screening tools for depression and anxiety (Cronbach alpha 0.86 and 0.91, respectively). The one-factor structure of the PHQ-9 and the GAD-7 was confirmed by the Confirmatory Factor Analysis. A cut-off of ≥10 for the PHQ-9 resulted in 71% sensitivity and 66% specificity recognizing students with increased risk for mood or anxiety disorder. For the GAD-7, a cut-off ≥9 resulted in 73% sensitivity and 70% specificity recognizing students at risk. The PHQ-9 was sensitive but not specific in recognizing students with depressive disorders. The sensitivity and specificity of the GAD-7 in differentiating students with generalized anxiety disorders were low.

**Conclusions:**

The PHQ-9 and the GAD-7 have sufficient formal psychometric properties, but their clinical utility as diagnostic tools for recognition of depressive and anxiety disorders in students is limited. Due to low specificity and high false positive rates, both scales are recommended only as an initial screening tool for recognition of subjects with increased risk of mental disorders, however positive cases should be later assessed using more comprehensive instruments.

## Introduction

University students often experience severe psychological distress and are at higher risk of mental disorders than other young people of their age in the general population [1, 2]. Although a university degree is considered as one of the resilience resources and is related to better health later in life, the process of education for some students might be stressful, as it requires adaptation to a novel environment, achievement of required standards, making important life choices, and planning for the future while stepping into adulthood [3, 4]. Meta-analysis by Ibrahim et al (2013) indicated that prevalence of depression in university students ranges from 10 to 85% with a weighted mean prevalence of 30.6% [5]. In a large sample of Italian students, 22% showed moderate depressive symptoms and 12% had serious and very serious symptoms; a moderate level of anxiety was observed in 19.7% of students, and in 7.5% the level of anxiety was extremely high [6]. In Australian students, the prevalence of major depression was 7.9%, and generalized anxiety disorder was 17.5% [2]. In a sample of 148 Canadian students, 39.5% had symptoms of moderate to severe depression, 23.8% had symptoms of moderate to severe anxiety and 80.3% had symptoms of moderate to high stress with no significant differences between males and females [3].

Depression and anxiety disorders may have both immediate and delayed negative outcomes, including decreased academic performance, interpersonal problems, underachievement and decreased quality of life [2, 7]. Depression is also related to increased risk of suicide in students [8, 9]. Thus, reliable, and valid instruments are needed for assessment of depressive and anxiety symptoms for timely recognition of need for psychological support and offer appropriate help for students [10]. The Patient Health Questionnaire-9 (PHQ-9) and the Generalized Anxiety Disorder Questionnaire-7 (GAD-7) are two short screening scales that are widely used for this purpose [11, 12].

The PHQ-9 and the GAD-7 are scales from a larger Patient Health Questionnaire (PHQ) created to measure depressive and anxiety symptoms. A full PHQ is a screening tool for criteria-based diagnoses of various mental disorders in primary care [13].

The PHQ-9 is a nine item scale consisting of the actual 9 criteria upon which the diagnosis of *Diagnostic and Statistical Manual of Mental Disorders (DSM)*, 4^th^ edition (DSM-IV) depressive disorders was based [14]. However, it is also comparable with more recent version of DSM, 5^th^ edition (DSM-5) [15]. The PHQ-9 is useful in making clinically meaningful classification of depressive symptomology; it also provides continuous scores for assessment of severity of depression [15]. The scale is shown to have high internal consistency and high test-retest stability [14]. Currently, it is one of the most frequently used and most validated measures of depression worldwide, which has been translated into over 70 languages [16]. The PHQ-9 is recognized as a valid and reliable instrument for screening of depression in the general population [10, 17], in clinical settings [18–21] and the university context [22, 23]. It has sufficient concordance with recognized clinical measures of depression such as the Composite International Diagnostic Interview, CIDI [10], the Structural Clinical Interview for DSM Disorders, SCID [24] and the MINI-International Neuropsychiatric Interview, M.I.N.I. [25]. Assessment can be performed using traditional pencil and paper forms as well as more modern means like smartphones [26] or online screening [27, 28]. The PHQ-9 demonstrates measurement invariance across sociodemographic subgroups of gender, ethnicity and education [29, 30], and, although some cultural differences are reported on individual items, results of summary scores are generally comparable across cultures [31].

The GAD-7 is a seven item self-report scale, specifically linked to the DSM-IV criteria of generalized anxiety disorder (GAD) [32]. Although targeted at assessment of generalized anxiety, the GAD-7 was also shown to be a valuable screening tool for assessment of other anxiety disorders, including panic, social anxiety, and posttraumatic stress disorders [13]. The GAD-7 displays high internal consistency and good convergent validity [32]. According to its authors, a score of 10 or greater on the GAD-7 represents a reasonable cut-off point for identifying cases of GAD [32]. The cut-off points of 5, 10, and 15 might be interpreted as representing mild, moderate, and severe levels of anxiety on the GAD-7 [32]. The GAD-7 is very widely used in clinical and nonclinical settings, including student research [1,2]. Although the GAD-7 is less researched than the PHQ-9, it has also been validated in various cultures [33, 34].

Despite numerous advantages, the PHQ-9 and the GAD-7 have several issues that need to be considered when applying these instruments in a different cultural and social context. First, there are mixed findings regarding the factor structure of the PHQ-9 and the GAD-7 [27, 35]. Some studies report an unidimensional structure of the PHQ-9 [20, 29, 36], other studies found that the two-dimensional structure of psychological and somatic symptoms demonstrate a better fit [31, 37]. Some studies also reported that the PHQ-9 and the GAD-7 are highly intercorrelated and can hardly be analyzed as two independent measures [38]. Teymori et al found that the PHQ-9 and the GAD-7 were two parts of a single general factor and shared one common domain of negative affectivity and negative information processing [38]. Recently a new measure, the PHQ-ADS, combining the PHQ-9 and the GAD-7 into a single score, was proposed as a measure of depressive and anxiety related symptoms [39].

Another important question is related to optimal cut-off values for the PHQ-9 and the GAD-7. Studies in different settings demonstrate that recommended cut-offs might need to be adjusted for certain populations [40]. For example, Munoz-Navaro et al. (2017) found that in Spanish primary care patients a cut-off value of 12 performed better while screening for major depressive disorder confirmed by SCID-I interview than the recommended cut-off of 10 [24]. In a study of 169 adult ambulatory patients with various degrees of depression, a cut-off of 8 was found to be optimal for recognizing major depressive disorder according to the M.I.N.I [21]. Similar results were found in a large sample of Latvian patients [25], while in a sample of university students the cut-off for minor depressive disorder was 5 [41]. Cut-off values might need to be revised due to sensitivity and specificity issues in certain populations. For example, meta-analysis by Levis et al indicates that PHQ-9 might be less specific for younger patients than for older ones [11].

Sensitivity and specificity problems were also reported for the GAD-7 [35]. For example, diagnostic meta-analysis by Plummer et al (2015) revealed that optimal sensitivity-specificity balance is more frequently achieved using lower cut-of scores than proposed by scale’s authors [12]. Similarly, a study in a big sample of various psychiatric patients a cut-off of 8 resulted in most precise classification of patients with anxiety disorders [42].

In this study we aim to evaluate psychometric properties of the PHQ-9 and the GAD-7 in a sample of Lithuanian students, specifically to investigate reliability, factor structure, criterion validity, sensitivity and specificity of the PHQ-9 and the GAD-7. This psychometric evaluation is important while judging diagnostic value of these instruments for recognition of depressive and anxiety disorders. To best of our knowledge psychometric properties of the PHQ-9 and the GAD-7 were never systematically studied in Lithuanian student population, thus this study adds to the development of evidence-based students assessment practice in Lithuania.

## Materials and methods

### Participants and procedures

This study was a part of a larger observational cross-sectional web-based survey investigating mood, internet use and gaming behavior and quality of life in Lithuanian students [43].

The study protocol was approved by the Bioethics Committee of the Lithuanian University of Health Sciences and was implemented in line with the principles outlined in the Declaration of Helsinki. To ensure participants’ anonymity, no questions were given that would compromise their identity. An online consent form was provided for each participant before starting the survey. A survey website and answer data were hosted on secured servers of the Lithuanian University of Health Sciences.

The study invitation with a link to an online survey was disseminated through social media, university websites and university e-mails. Subjects were included in the study if they indicated that they were university students and were 18 years or older. Data was gathered from May of 2020 to June of 2021.

**Fig 1:**
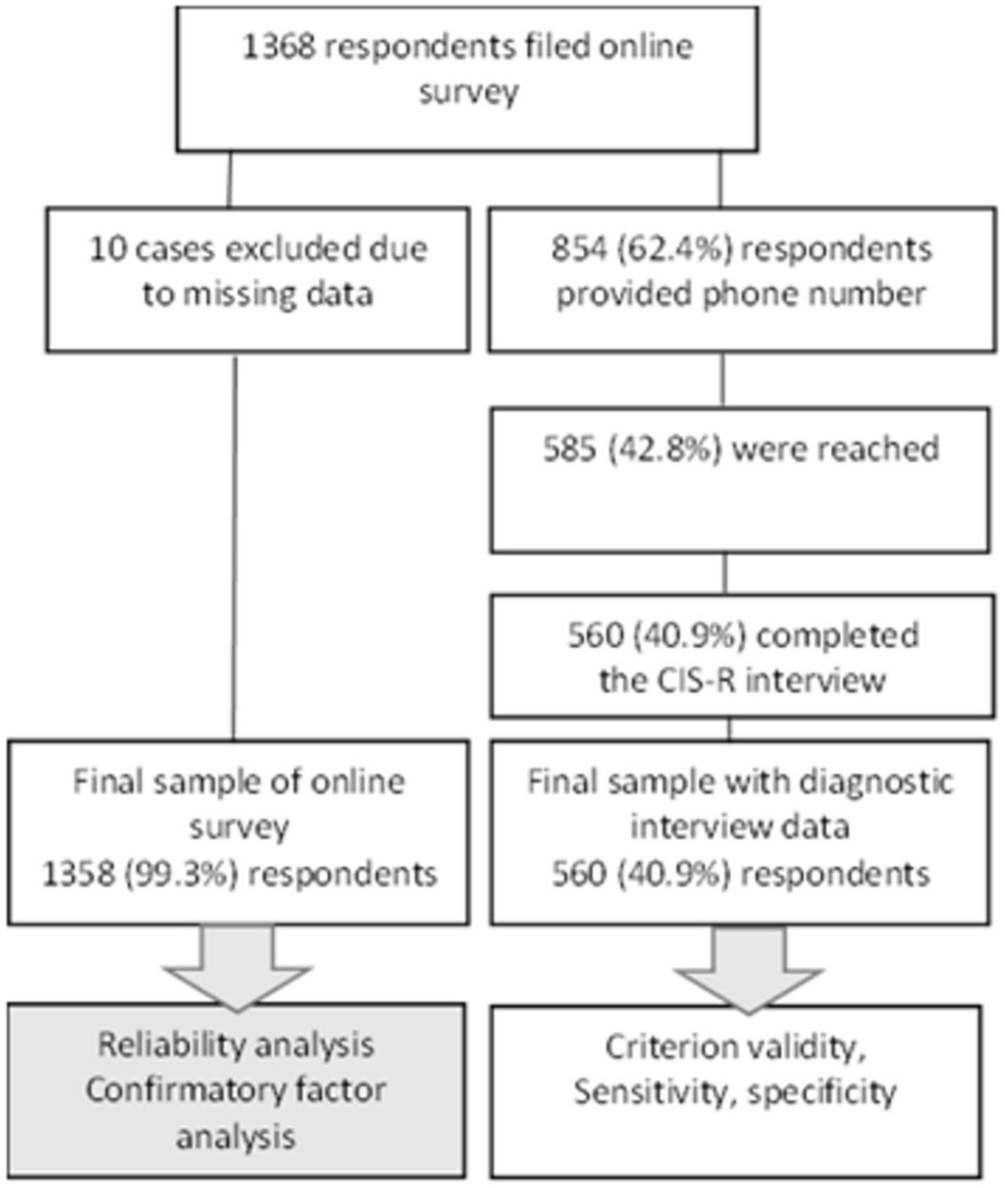
Diagram of participants’ flow.

In total, 1368 students from various universities in Lithuania responded to the invitation and participated in an anonymous online survey. After the completion of the online survey participants were asked to provide their phone number if they agreed to be contacted by the research team for an additional interview. Overall, 854 (62.4% of a total sample) students provided a valid phone number for the additional interview. All the participants who provided phone numbers were called by research team members, and if a subject was reached and agreed to participate, structured diagnostic interview using The Clinical Interview Schedule-Revised (CIS-R) was conducted to verify presence of depressive and anxiety symptoms. To ensure the smallest possible drop-out rate, a second attempt was made to reach if a subject was not reachable after the first call; if subjects was still not reachable after the second call, a text message with invitation to participate in the study was sent. If a participant was not able to participate in the interview because of the inappropriate time, place, busy schedule etc. at the time of the call, the new time for the interview was offered and negotiated. In sum, 585 (42.8% of total sample) students were finally reached, and 560 of them (40.9% from the total sample and 65.6% from those who agreed to provide a phone number) completed the full CIS-R interview. Structured information on participants flow is provided in Fig 1.

Despite the high drop-out rate, online survey and interview samples remained comparable regarding proportion of males and females, distribution of age, field of study and the PHQ-9 and the GAD-7 scores. A description of the samples is provided in Table 1.

**Table 1:** Demographic and clinical characteristics of online and interview samples.

	Total online sample (N = 1358)	Interview subsample (N = 560)
	*Mean (SD)*, *N (%)*	
Gender		
*Male*	221 (16.3%)	99 (17.7%)
*Female*	1134 (83.5%)	459 (82.0%)
*Other*	3 (0.2%)	2 (0.4%)
Age	22.5 (±4.8), range 18–57	22.7 (±5.0), range 18–56
Field of study		
*Mathematics and informatics*	107 (7.9%)	43 (7.7%)
*Physical and life sciences*	127 (9.4%)	62 (11.1%)
*Engineering and technologies*	168 (12.4%)	51 (9.1%)
*Health and veterinary sciences*	377 (27.8%)	151 (27.0%)
*Agricultural sciences*	23 (1.7%)	7 (1.3%)
*Social sciences*	385 (28.4%)	174 (31.1%)
*Humanities*	147 (10.8%)	62 (11.1%)
*Arts*	21 (1.5%)	8 (1.4%)
*Sports*	3 (0.2%)	2 (0.4%)
PHQ-9	9.8 (±5.7), range 0–27	9.7 (±5.5), range 0–26
GAD -7	8.2 (±5.5), range 0–21	8.2 (±5.4), range 0–21

Structured diagnostic interview using the CIS-R was conducted by three professional psychologists (PA, JB, JGS), trained to perform CIS-R assessment. Interviewers were blind to data of online assessment to ensure independence of self-report and interview assessments. The CIS-R asks respondents about symptoms experienced during one month period, thus it was aimed to conduct the interview within the these reference time limits to ensure overlap of reported symptoms in online and interview assessments. However, this was not always possible to implement, because respondents frequently could not talk at the times of the first telephone call and new time for the interview had to be negotiated. Mean interval between online assessment and phone interview was 23.5 (± 25.1) days. Average duration of the interview was 21.9 (SD 8.3) minutes. If during the interview a student expressed serious mental health concerns or suicidal ideation, psychological counselling was immediately offered and information about mental health services in the community was provided. Students were also offered the possibility to discuss their assessment results and to ask questions after the completion of the interview. Most students appreciated this opportunity to discuss their mental health needs and felt positive about the interview.

### Measures

The PHQ-9 is a brief self-report tool for screening the presence of depression and its symptom severity [14]. Possible response options range from “Not at all” to “Nearly every day”. The total scores range from zero (0) to 27 with higher scores indicating more expressed depressive symptoms. Lithuanian language version which is available at the PHQ Screeners website https://www.phqscreeners.com/ was used for current research. Although this instrument is widely used for research purposes in Lithuanian, national cut-of values for general population are not established [44]. For this reason, in current study, we used cut-points of 5, 10 and 15 as indicators of mild, moderate and severe depression, as suggested by the questionnaire authors [39].

The GAD-7 is a seven item self-report instrument that is used to assess the severity of GAD and general anxiety symptoms [32]. Each item asks the individual to rate the severity of his or her symptoms over the past two weeks using a four-point Likert scale with possible responses ranging from “Not at all” to “Nearly every day”. The total scores range from zero (0) to 21 with higher scores indicating more expressed anxiety symptoms. Lithuanian language version which is available at the PHQ Screeners website https://www.phqscreeners.com/ was used for current research. Although the GAD-7 was extensively used in various studies in Lithuania, comprehensive psychometric evaluation of this questionnaire was not performed. In the current study we used cut-points of 5, 10 and 15 as indicators of mild, moderate and severe anxiety, as proposed by scale authors [32].

The Clinical Interview Schedule-Revised (CIS-R) is a standardized clinical interview aimed at measuring minor psychiatric disorders [45]. The CIS-R was used as a criterion for assessment of diagnostic validity of the PHQ-9 and the GAD-7 in this study. Results of the CIS-R can be analyzed at different levels.

1. The CIS-R measures the nature and severity of neurotic symptoms that were present for at least seven days in 14 different symptoms groups, including somatic symptoms, health worries, panic, compulsive behaviors, obsessive thoughts, phobias, irritability, worry, anxiety, forgetfulness / concentration problems, fatigue, sleep, depression, and depressive thoughts. Each of the CIS-R sections contains a series of questions designed to score the specific psychiatric symptom groups. All the answers range from zero (0) to four (4), except for depressive thoughts scores that range from zero (0) to five (5). Higher scores reflect higher levels of symptomatology. Symptoms with the score of two (2) and more are considered significant. The CIS-R symptoms groups were used as the criterion validity measure in current study.
2. The CIS-R produces a total score ranging from zero (0) to 57, where a total score of ≥ 12 shows a significant level of distress [46]. The cut-off value of 12 has been reported to have sensitivity of 87.9% and specificity of 96.2% in detecting psychiatric disorders [46]. The Total CIS-R score was used to evaluate sensitivity and specificity of the PHQ-9 and the GAD-7 to possible nonspecific psychiatric morbidity.
3. The CIS-R also generates diagnostic categories in accordance with the *International Classification of Diseases*, 10^th^ edition (ICD-10) criteria, where diagnostic conditions need to be present for at least two weeks. Diagnostic categories include: no diagnosis; mixed anxiety and depressive disorder (MADD); GAD; obsessive-compulsive disorder; specific (isolated) phobia; social phobia; agoraphobia; panic disorder; mild/moderate/severe depressive episode (DE). Diagnostic categories of GAD, MADD and DE were used in current study to evaluate sensitivity and specificity of the PHQ-9 and the GAD-7 for recognition of specific psychiatric problem.

Decision to employ the CIS-R in current study was based on several important advantages of this interview. Firstly, this is a fully structured interview which helps to ensure high compatibility between interviewers. The CIS-R includes a computer algorithm (PROgrammable Questionnaire SYstem, PROQSY), which guides interview questions and enables generation of ICD-10 diagnosis thus minimizing observer bias [47]. The CIS-R might be administered not only by psychiatrist but also by other trained health care professionals [47]. Comparability of CIS-R results and clinical diagnosis was widely investigated, and studies suggest that the instrument remains valid across a number of cultural settings and age groups [48–50]. The CIS-R is one of the few clinical instruments that can be used free of charge in clinical research. Permission to use this interview in current study was obtained from the authors (G.Lewis, personal communication); forward translation to Lithuanian language an back translation to English was performed while translating the interview and the scoring program. Translations were performed by bilingual specialists with experience in the mental health domain. All interviewers participated in a one-hour standardization session including telephone interview to ensure comparability of interview administration.

Current study was implemented in the contexts of the COVID-19 pandemic. To evaluate possible effect of the lockdown and other pandemic induced life changes on students’ depression and anxiety scores, a question about mood change was included in the study “*How did COVID-19 situation influence your mood*, *how you feel in general*?*”*. Response options ranged from: 1—My mood decreased very significantly to 5—My mood significantly improved.

### Statistical analysis

Data was analyzed using IBM SPSS Statistics for Windows (version 20) and SPSS AMOS (version 20) (IBM Corp., Armonk, NY, USA). Before conducting the analysis, the items of the PHQ-9 and the GAD-7 were screened for missing values and normality. The normality of the distributions was assessed at the univariate and multivariate levels. Internal consistency was examined using corrected item-total correlations and Cronbach’s alpha coefficient. Correlations were analyzed using Pearson’s correlation coefficient and Spearman’s r correlation coefficient. Differences between diagnostic groups were assessed applying one-way ANOVA. The powers of discrimination, sensitivity and specificity of the PHQ-9 and the GAD-7 were evaluated applying the ROC analysis. The factorial structure of the PHQ-9 and the GAD-7 were valuated by performing confirmatory factor analysis (CFA) using maximum likelihood estimation. The model fit was evaluated using the Chi-square test and the following indices: standardized root mean square residual (SRMR), goodness of fit index (GFI), comparative fit index (CFI), and root mean square error of approximation (RMSEA).

## Results and discussion

### Study samples description

Demographic characteristics of the study samples are provided in Table 1. Females were significantly overrepresented both in online and interview samples (83.5% and 82% respectively). Average age of participants was 23 years, age range was relatively wide, from 18 to 56–57 years both in online and interview samples. Both samples represented wide range of study disciplines; students from health and veterinary sciences and social sciences constituted slightly more than a half of all the participant is both samples. Because study was performed in contexts of the COVID-19 pandemic, it was important to evaluate whether students from health sciences do not differ in their expressed distress levels, as in many countries medical students were recruited to work in the red zones. Statistical analysis did not reveal significant differences in the PHQ-9 or the GAD-7 between students from health disciplines and students from other disciplines (for the PHQ-10 mean 9.6 (SD 5.4) and 9.9 (SD 5.8); p = 0.39; for the GAD-7 mean 8.1 (SD 5.2) and 8.2 (5.6); p = 0.61).

### Prevalence of depressive and anxiety symptoms

When cut-offs of ≥5; ≥10 and ≥15 proposed by the PHQ-9 and the GAD-7 authors were used, prevalence of depressive and anxiety symptoms in Lithuanian students were extremely high. Only 19% of students reported no clinically significant depressive symptoms, while more than 45% scored in the moderate to severe range (Table 2). Severe anxiety was slightly less prevalent, almost 30% of students reported no clinically significant symptoms of anxiety, while more than 38% of students reported moderate to severe anxiety.

**Table 2:** Prevalence of depressive and anxiety symptoms in Lithuanian university students.

	*Online sample (N = 1358)*			
	None	Mild	Moderate	Severe
Prevalence of depressive symptoms according to PHQ-9 cut-offs (5/10/15)	259 (19.1%)	485 (35.7%)	338 (24.9%)	276 (20.3%)
Prevalence of anxiety symptoms according to GAD-7 cut-offs (5/10/15)	398 (29.3%)	440 (32.4%)	315 (23.2%)	205 (15.1%)
***CIS-R interview subsample (N = 560)***				
Prevalence of clinically significant psychiatric symptoms according to CIS-R cut-off (CIS-R≥12)				175 (31.3%)
Probable primary ICD-10 diagnosis according to CID-R interview				
Specific (isolated) phobia	8 (1.4%)			
Agoraphobia	2 (0.4%)			
Obsessive-compulsive disorder	1 (0.2%)			
Social Phobia	0 (0%)			
Panic Disorder	0 (0%)			
Mixed anxiety and depressive disorder	68 (12.1%); of these, cases with mild symptoms 59 (10.5%)			
Generalized anxiety disorder	54 (9.7%); of these, cases with mild symptoms 47 (8.4%)			
Depressive episode	81 (14.5%); of these, mild—28 (5.0%); moderate—35 (6.3%); severe—18 (3.2%)			
Number of respondents meeting criteria for more than one mood or anxiety disorder according to CID-R interview				118 (21.1%)

According to the CIS-R clinical interview cut of ≥12, 31.3% of students exhibited symptoms that indicated possible psychiatric morbidity (Table 2). The most prevalent problem was depression; 14.5% of students met the criteria for depressive episode (DE). Symptoms of mixed anxiety and depressive disorder (MADD) were observed in 12.1% of students, although in most cases the disorder was mild. 9.7% of students reported symptoms of GAD. None of the students met the criteria for social phobia or panic disorder as a primary diagnosis. 118 respondents (21.1%) met for more than one mood or anxiety disorder according to the CIS-R interview 118 (21.1%).

29.5% of students reported some form of suicidal ideations (the PHQ-9 item 9 “Thoughts that you would be better off dead, or of hurting yourself”). Specifically, 246 (18.1%) respondents reported having thoughts about being dead or hurting him/herself for several days, 93 (6.8%) more than half the days and 62 (4.6%) almost daily.

During the CIS-R interview 25% of respondents reported symptoms related to increased suicidal risk. 94 (16.8%) reported hopelessness without active suicidal thoughts, 28 (5.0%) subjects reported feeling that life isn’t worth living, 15 (2.7%) reported active suicidal thoughts and 3 (0.5%) reported having suicidal plans. In all cases when suicidal ideations were reported, psychological help and guidance were offered.

210 (15.5%) of students reported very significant decrease in their mood due to COVID-19 pandemic, 567 (41.8%) reported slight negative changes, while 581 (42.9%) reported no change or even improvement in their mood. Mood changes due to COVID-19 pandemic were weakly but statistically significantly correlated to the PHQ-9, the GAD-7 and the CIS-R scores (Spearman rho, -0.355, -0.345 and -0.138, p<0.01, accordingly), indicating that all measures were significantly affected by increased situational distress; however self-report measures were affected to a higher degree.

### Reliability of the PHQ-9 and GAD-7

Reliability analysis showed that the PHQ-9 items were highly consistent, Cronbach alpha = 0.86. However, the inter-item correlations were weak to moderate (mean r = 0.40, min-max = 0.25–0.64).

The GAD-7 also demonstrated good internal consistency, Cronbach alpha = 0.91. The inter-item correlations were in the moderate-high range (mean r = 0.60, min-max = 0.48–0.77).

### Factor structure of the PHQ-9 and GAD-7

Assessment of the univariate and multivariate normality was performed for the items used in the CFA models. Multivariate outliers of the PHQ-9 and the GAD-7 were removed using the Mahalanobis distance measure (critical value 27.9 and 24.3, respectively, Chi-squared test p<0.001). Multivariate kurtosis and critical ratio were 12.70 and 16.46 for the PHQ-9 items; and 8.02 and 13.03 for the GAD-7 items implying a moderate multivariate normality in this sample.

**Fig 2:**
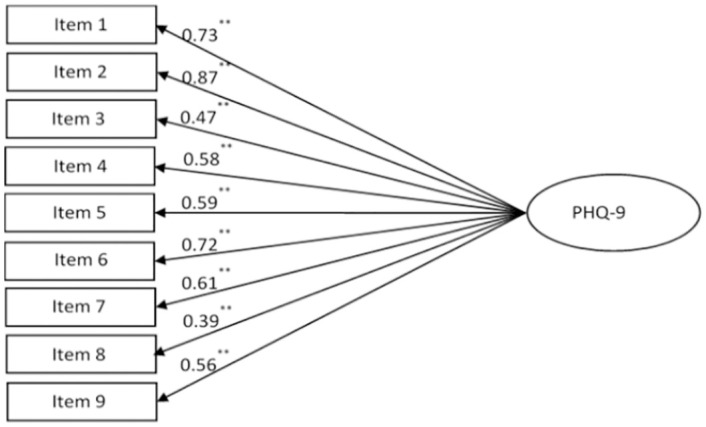
One-factor structure of the PHQ-9 confirmed by CFA: Chi-square value = 25.418, df = 16, p = 0.063, SRMR = 0.0151, GFI = 0.996, CFI = 0.998, RMSEA = 0.021. Path coefficients are standardized (**p<0.001).

The results of the CFA analysis supported the one-factor structure of the PHQ-9 and demonstrated good fit: Chi-square value = 25.418, df = 16, p = 0.063, SRMR = 0.0151, GFI = 0.996, CFI = 0.998, RMSEA = 0.021 (Fig 2). Items had statistically significant factor loadings varying from 0.39 up to 0.87 (p<0.001). The correlations between residuals of indicators were low (from -0.311 up to 0.359).

We also tested a two-factor model of the PHQ-9 (Table 3), testing the hypothesis that Items 1, 2, 6, 7, 9 belong to the psychological factor, and items 3, 4, 5, 8 belong to the somatic factor. However, the two-factor model was not supported by CFA (Chi-square value = 117.181, df = 19, p<0.001). The results showed that factors were highly correlated (r = 0.82, p<0.001) and suggested that PHQ-9 items form one factor.

**Table 3:** Fit of the CFA models tested in the study.

	Fit Values						Factor correlations
Models	χ^2^(*df*)	*p-value*	CFI	GFI	RMSEA (90% CI)	SRMR	
PHQ-9 1-F	25.418(16)	0.063	0.998	0.996	0.021 (0.000–0.036)	0.0151	-
PHQ-9 2-F	117.181(19)	<0.001	0.977	0.957	0.062 (0.052–0.073)	0.0289	0.82
GAD-7 1-F	11.612(8)	0.169	0.999	0.998	0.018 (0.000–0.040)	0.0085	-
GAD-7 2-F	115.477(13)	<0.001	0.983	0.976	0.077 (0.064–0.090)	0.0241	0.95

F = factor; χ2 = Chi-square; *df* = degrees of freedom; CFI = comparative fit index; GFI = goodness of fit index; RMSEA = root mean square error of approximation; CI = confidence interval; SRMR = standardized root mean square residual.

**Fig 3:**
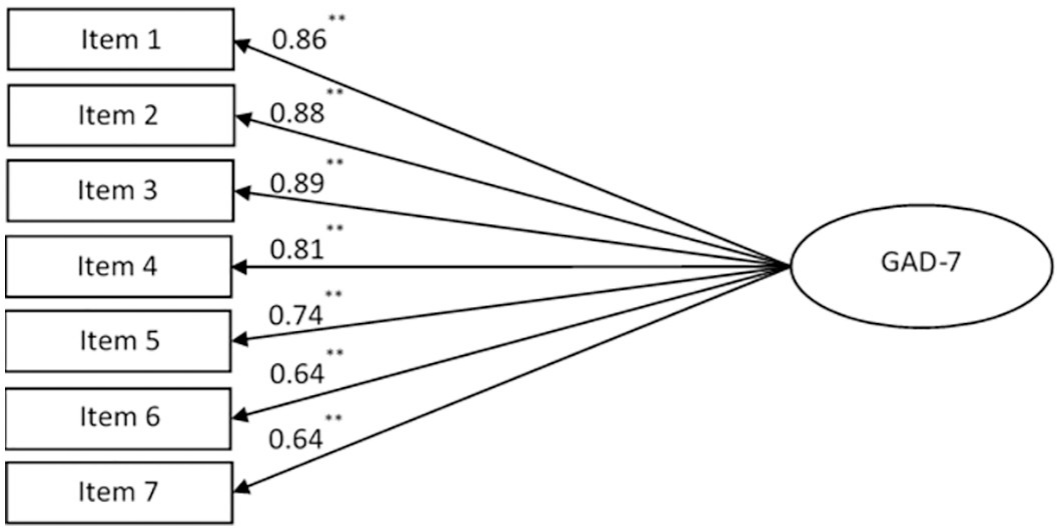
One-factor structure of the GAD-7 confirmed by the CFA: Chi-square value = 11.612, df = 8, p = 0.169. Path coefficients are standardized (**p<0.001).

CFA also confirmed a one-factor structure of the GAD-7 (Fig 3) and indicated good fit of the unifactorial model: Chi-square value = 11.612, df = 8, p = 0.169, SRMR = 0.0085, GFI = 0.998, CFI = 0.999, RMSEA = 0.018. The correlations between residuals of indicators were low (from -0.297 up to 0.144). Factor loadings were high (0.64–0.89) and statistically significant (p<0.001).

Two-factor model of the GAD-7 was not confirmed (Table 3): Chi-square value = 115.477, df = 13, p<0.001. The two factors integrating four cognitive-emotional items 1, 2, 3, 7, and three somatic items 4, 5, 6, were highly correlated (r = 0.95; p < 0.001). The results indicated that the GAD-7 is better represented as a single construct.

### Criterion validity of the PHQ-9 and GAD-7

To evaluate criterion validity of the PHQ-9 and GAD-7, correlations between summary scores of these scales and CIS-R symptom groups were analyzed (Table 4). Scores from the PHQ-9 and the GAD-7 significantly correlated with most of CIS-R symptom groups. As could be expected, slightly stronger correlations were observed between the PHQ-9 scores and suicidal ideation, poor concentration, fatigue, sleep problems, and depressive ideas. The GAD-7 was slightly stronger than PHQ-9 related to worry over physical health, irritability, phobic symptoms, general worries, and compulsive behavior. However, no significant differences were observed in correlations with the CIS-R symptoms groups of depression and anxiety which may raise a question about discriminant validity of PHQ-9 and GAD-7.

**Table 4:** Correlations between the PHQ-9, GAD-7 and the CIS-R symptoms groups.

CIS-R symptoms groups scores	PHQ-9 Total score	GAD-7 Total score
Suicide ideation	0.414**	0.363**
Somatic symptoms	0.287**	0.295**
Worry over physical health	0.196**	0.234**
Irritability	0.210**	0.262**
Poor concentration	0.360**	0.293**
Fatigue	0.391**	0.349**
Sleep problems	0.349**	0.272**
Depression	0.414**	0.415**
Depressive ideas	0.447**	0.419**
Phobias	0.129**	0.166**
Worry	0.319**	0.419**
Anxiety	0.336**	0.396**
Panic	0.143**	0.163**
Compulsions	0.088*	0.143**
Obsessions	0.158**	0.143**

*Spearman rho<0.05,

**Spearman rho<0.01.

The PHQ-9 and the GAD-7 scores were also compared in subgroups of students, who met criteria for probable ICD-10 diagnosis in the CIS-R. Four groups based on primary probable diagnosis were created for this analysis: (1) No ICD-10 diagnosis (N = 346 (63.0%)); (2) MADD (N = 68 (12.4%)); (3) GAD (N = 54 (9.8%)); (4) DE (N = 81 (14.8%)). ANOVA was used to compare the PHQ-9 and the GAD-7 scores between those groups controlling for possible effect of age and gender.

Results showed that there was a significant effect of having met the criteria of anxiety or depressive disorder on the PHQ-9 scores, F(3, 546) = 24.00, p<0.001; students, who scored higher on the PHQ-9 were more frequently diagnosed with DE, MADD or GAD. No significant agender effect was observed F(1, 546) = 0.32, p = 57. However age was related to the PHQ-9 scores, F(1, 546) = 6.05, p = 0.02.

A similar effect of diagnosis was observed for the GAD-7 scores, F(3, 546) = 20.61, p<0.001. Students who were diagnosed as having increased risk of DE, GAD or MADD disorder according to the CIS-R interview, tend to score higher on the GAD-7. No significant effect of age and gender was observed: age F(1, 546) = 0.30, p = 0.58, gender F(1, 546) = 0.24, p = 0.62.

### Sensitivity and specificity of the PHQ-9 and GAD-7

Several criteria were used while calculating sensitivity and specificity of the PHQ-9 and the GAD-7. Firstly, the area under the curve (AUC) was calculated for both measures, using a non-specific indicator of possible psychiatric morbidity, CIS-R≥12. Next, we analyzed AUC of the PHQ-9 and the GAD-7 in specific diagnostic subgroups of MADD, GAD and DE.

**Fig 4:**
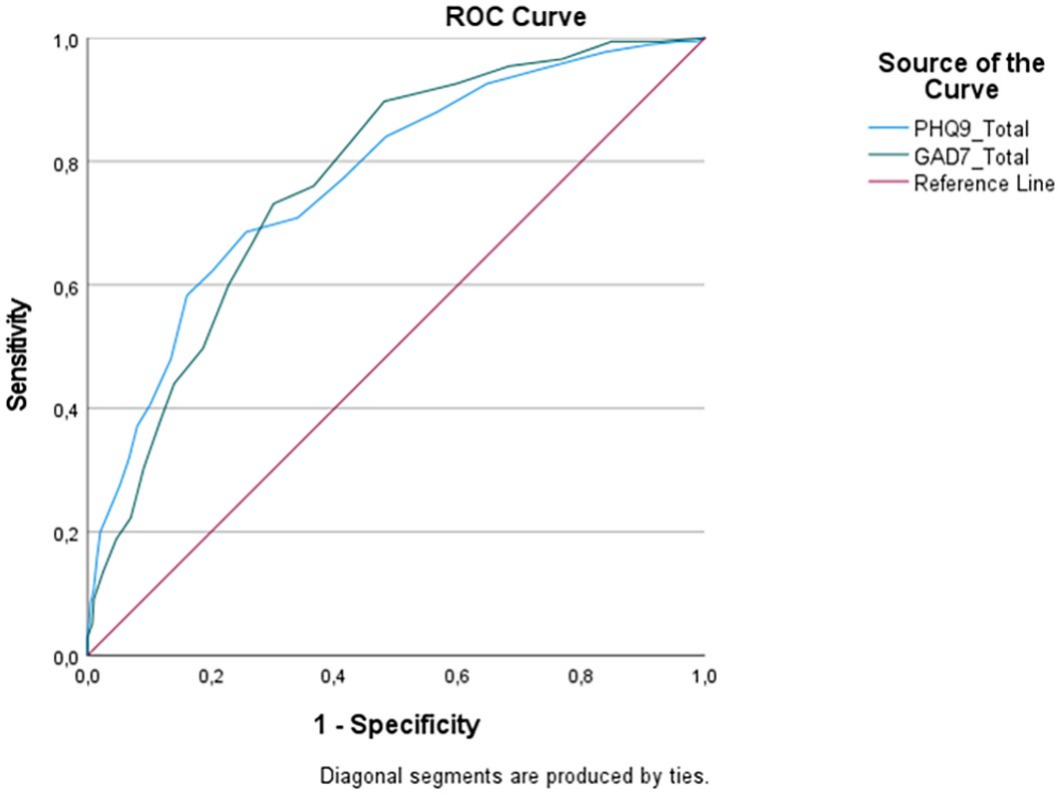
ROC curve for predicting risk of anxiety and depressive disorders in students using the PHQ-9 and the GAD-7 total scores.

Analysis revealed that the PHQ-9 (AUC = 0.77, p<0.001, 95% C.I. 0.73–0.81) and GAD-7 (AUC = 0.77, p<0.001, 95% C.I.0.73–0.81) share similar moderate discriminatory power recognizing students possibly having a mental disorder. Results indicated that using a cut-off of ≥10 for the PHQ-9 would result in 71% sensitivity and 66% specificity recognizing students at risk. For the GAD-7, a more optimal cut-off was slightly lower (of ≥9) which resulted in 73% sensitivity and 70% specificity (Fig 4).

Discriminant power of the PHQ-9 (AUC = 0.62, p = 0.001, 95% C.I.0.55–0.69) and the GAD-7 (AUC = 0.65, p<0.001, 95% C.I.0.59–0.71) for recognition of MADD subgroup was weak and it was difficult to find the optimal sensitivity and specificity ratio. For example, a cut-off of ≥6 on the PHQ-9 would have 88% sensitivity, but only 29% specificity recognizing MADD.

Similar problems were observed when predicting GAD cases. Contrary to expectations, the GAD-7 had no significant advantage recognizing GAD cases when compared to the PHQ-9: the PHQ-9 AUC = 0.66, p<0.001, 95% C.I.0.59–0.72; the GAD-7 AUC = 0.68, p<0.001, 95% C.I.0.62–0.75. Significant problems with specificity were observed in both measures. For example, a cut-off of ≥6 in the PHQ-9 would result in 93% sensitivity, but only 29% specificity. 7 or more points in the GAD-7 would help to identify 85% of GAD cases correctly, but 48% of cases would be false positives.

The PHQ-9 slightly outperformed the GAD-7 when discriminating cases of DE. The PHQ-9 demonstrated moderate discriminant power (AUC = 0.77, p<0.001, 95% C.I.0.72–0.84), while the GAD-7 remained in the low-moderate range (AUC = 0.75, p<0.001, 95% C.I.0.69–0.80). Cut-off of ≥10 in PHQ-9 resulted in 83% sensitivity and 61% specificity. For the GAD-7, cut-off of ≥9 demonstrated 82% sensitivity and 62% specificity in recognizing cases of DE.

## Discussion

In this study we aimed to evaluate psychometric properties of the PHQ-9 and the GAD-7 in a sample of Lithuanian students, to investigate the factor structures of these instruments and to determine their diagnostic value for recognition of depressive and anxiety disorders in a student population. Psychometrically sound screening instruments are important for mental health specialists working in college and university settings, because many studies report high prevalence of depressive and anxiety symptoms in students [1, 3, 5, 7]. Our data not only supports these findings, but also illustrates that prevalence rates are highly dependable on measures that were used for the assessment. According to the PHQ-9 and the GAD-7, 45% of students met the criteria for moderate to severe depressive symptoms and 38% of students reported moderate to severe anxiety. According to the CIS-R clinical interview, 12.1% of students in our sample met the criteria for MADD, 14.5% of students met criteria for DE and 9.7% for GAD. These rates are significantly lower than those obtained from the PHQ-9 and the GAD-7, but still much higher than the total 14% prevalence of anxiety disorders and depression in the Lithuanian general population as was reported by the Eurobarometer survey. Almost 30% of students reported thoughts of better being dead or of hurting themselves and 4.6% of students reported them on daily basis. These findings illustrate that mental health needs of young people should be addressed properly. In the face of COVID-19 pandemic this might be even more important. Results of our study indicate that more 15% of university students report significant decrease and 41% report slight decrease of their mood due to pandemic situation. Thus, simple, short, and valid measures for identification of psychological distress symptoms become very important in identification of students at risk.

In line with previous findings, the internal consistency of the PHQ-9 and the GAD-7 was high, 0.86 and 0.91 respectively. Both measures correlated significantly with symptoms related to depression and anxiety measured by CIS-R. Moderate correlations between the PHQ-9 and other criterion measures were also reported in other studies [20]. Moderate correlations should not be considered as a limitation of the instruments, but rather an indicator of a unique informational value of these scales.

With regards to factorial validity, CFA indicated one-factor structure of the PHQ-9 and the GAD-7. The unifactorial structure of these scales was reported by scales’ authors as well as by many other studies [13, 20, 31, 32, 36]. However, in our study the PHQ-9 and the GAD-7 were significantly correlated and an overlap between their items was observed. These findings indicate low discriminant validity of these measures and suggest that using both instruments may have low additive value when compared to application of a single instrument. Significant overlap between measures of depression and anxiety is not a new problem, especially for self-report measures [38]. Although some studies have provided the evidence for the distinctiveness of the two constructs [32], other authors argue that a hierarchical model of combined depressive and anxiety factors has higher ecological validity, as both depression and anxiety are parts of the mood disturbance and share many similar communalities [38]. Recently, Kroenke et al. combined items of the PHQ-9 and the GAD-7 that proposed a composite measure of depression and anxiety called the Patient Health Questionnaire Anxiety and Depression Scale (PHQ-ADS) [39]. However, according to the authors, summary score does not override individual value of the PHQ-9 and the GAD-7 [39]. Our study partially supports these results.

Findings regarding convergence of results between the PHQ-9, the GAD-7 and diagnostic categories of ICD-10 disorders diagnosed by the CIS-R are mixed. The discriminant power of both instruments was lower in our study than reported in previous research [11, 28, 51]. In general, both instruments were sensitive enough for classification of students at risk of a mental disorder, however, ability to discriminate between subgroups of anxiety and depressive disorders was below optimal. Low sensitivity and specificity of the PHQ-9 and the GAD-7 was previously reported in other studies. For example, in the sample of Mozambique primary care patients the PHQ-9 cut-off value of ≥9 demonstrated low sensitivity (46.5%) but satisfactory specificity (93.5%) [18]. In a sample of general Swiss general hospital patients, the PHQ-9 showed an acceptable level of specificity. However, its sensitivity in detecting major depression diagnosed by psychiatrist after extensive clinical interview was low, resulting in about 50% of false-negative results [52]. Appropriate sensitivity but suboptimal specificity of the GAD-7 was reported in a large Dutch sample of the general population [53]. Validity of the GAD-7 was not confirmed for anxiety screening in acute psychiatric settings [54]. In a study of Arabic speaking psychiatric outpatients, the PHQ-9 was sensitive but not specific in capturing depressive symptoms when compared to clinical diagnosis, whereas the GAD-7 was neither sensitive nor specific at capturing anxiety symptoms [33]. However, in student samples the PHQ-9 and the GAD-7 were reported to be valid and reliable [23, 28, 41].

Differences in accuracy of the PHQ-9 and GAD-7 might be related to language and cultural differences, criterion measure used and the timing of assessment. Although preliminary studies report only minimal influence of culture and ethnicity on the PHQ-9 results [36], some research suggests that differences in responding to specific items might be observed [31], and these subtle influences might impact the diagnostic properties of the scale. Possible cultural bias of the GAD-7 was also reported [35]. Thus, further studies of Lithuanian versions of the PHQ-9 and GAD-7 are needed for deeper investigation of item structure and diagnostic performance of the scales at item level.

Lower discriminant power observed in our study was likely related to timing of the two assessments. There was about three weeks’ interval between the self-report assessment using the PHQ-9 and the GAD-7 and the clinical interview using the CIS-R. Although in CIS-R a main time reference asking about presence of the symptoms was one month, and a significant overlap in reporting of symptoms between online assessment and interview was expected, there is a possibility that discrepancy between findings may reflect natural fluctuations of emotional state of the students. Self-report measures, like the PHQ-9 and the GAD-7, might be more sensitive to situational emotional states, than clinical interviews with robust framing and specification of symptom duration.

The type of interview might also be related to lower agreement between measures. Meta-analysis of Levis et al (2019) indicates, that greater concordance between the PHQ-9 and interview is achieved using semi-structured interviews, while using fully structured interviews concordance is lower due to strict interview rules that impede interviewer efforts to capture individualized experience of the subject which is reflected in a self-report [11]. The CIS-R is a fully structured interview. This sort of interview was intentionally chosen for the study to ensure good comparability of the data between three interviewers and because of better suitability for telephone assessment. However, a certain level of inflexibility should be acknowledged as a disadvantage in the diagnostic process.

Several additional limitations of this study should be acknowledged. Study was based on the convenience sampling of students from the universities of Lithuania; thus, the generalizability of the results should be considered with caution. Females were significantly overrepresented both in the online assessment and telephone interview, which may affect observed prevalence of depressive and anxiety symptoms. Data about gender differences in depression and anxiety prevalence in university students is mixed. Some studies report higher rates of mood disturbances in female students [2, 6], other studies found no gender differences [1, 3, 4]. Although no gender effect was found in our study, more gender balance sampling is recommended for future studies to clarify possible impact of gender on anxiety and depressive symptoms.

Only 62% of students provided their phone number for the additional interview assessment. It is possible that students who agreed to participate in the interview had certain motivation or personality traits, for example, were experiencing a high level of stress and were motivated by the desire for psychological help.

Although CIS-R is a well validated clinical diagnostic interview, it was translated into Lithuanian language for current study and conducted through the telephone. Research indicates that telephone assessment of the CIS-R is valid and comparable with face-to-face assessment [55]. However additional research of the Lithuanian version of the CIS-R, especially conforming proposed cut-off scores for symptoms severity and diagnostic categories is needed.

Current study was implemented in the contexts of COVID-19 pandemic. Our data indicates that more than 57% of students report decrease in their mood due to COVID-19 situation, and it seems that the PHQ-9 and the GAD-7 scores are more sensitive to such a situational distress than more strictly defined the CIS-R interview. Thus, situational factors might be at least partially related to lower sensitivity and specificity of the PHQ-9, and the GAD-7. However, the big sample size and comprehensive clinical interviews are the main strengths of this study.

## Conclusions

Depressive and anxiety symptoms are prevalent in Lithuanian students, and the PHQ-9 and GAD-7 are valuable instruments for recognition of students at increased risk of mental health problems. However, due to the low specificity, the PHQ-9 and GAD-7 are more suitable for initial screening rather than diagnostic assessment. More detailed psychological evaluations for students at risk are required for an accurate clinical diagnosis and treatment purposes.

## Supporting information

S1 Dataset (SAV)
